# Perspectives of program directors regarding candidate selection for the Saudi Board of Restorative Dentistry program

**DOI:** 10.3389/fmed.2026.1744123

**Published:** 2026-02-24

**Authors:** Ali Robaian AlQahtani, Mubashir Baig Mirza, Yazeed AlQahtani, Mona Tariq Aldaijy, Abdulrahman Almalki, Shahad AlBader, Rasha Alharthi, Lamya Alkheledan, Abdullah AlShehri

**Affiliations:** 1 Conservative Dental Sciences, College of Dentistry, Prince Sattam Bin Abdulaziz University, Al-Kharj, Saudi Arabia; 2 College of Dentistry, Prince Sattam Bin Abdulaziz University, Al-Kharj, Saudi Arabia; 3 Restorative Dentistry, Ministry of Health, Riyadh, Saudi Arabia; 4 Department of Prosthetic Dental Sciences, College of Dentistry, Prince Sattam Bin Abdulaziz University, Al-Kharj, Saudi Arabia; 5 Department of Clinical Dental Science, College of Dentistry, Princess Noura Bint Abdulrahman University, Riyadh, Saudi Arabia; 6 Private Practice, Riyadh, Saudi Arabia

**Keywords:** perspectives, program directors, residents, restorative dentistry, Saudi Arabia

## Abstract

**Introduction:**

Program directors (PDs) play a crucial role in selecting candidates for the Saudi Board of Restorative Dentistry (SBRD) program. However, their views on selection and possible differences in opinions based on candidate characteristics remain unelucidated.

**Methods:**

This study surveyed restorative dentistry PDs in Saudi Arabia using a pre-validated questionnaire consisting of 30 questions grouped into seven domains to gather views on the selection process for the SBRD program. Responses from 30 participants were analyzed using means and standard deviations. We examined differences in PD variables, such as sex and years of experience using Student’s *t*-tests. Variations related to geographic location, years of experience as a PD, and hospital setup were assessed using analysis of variance.

**Results:**

The top preferences of the PDs were working as service residents, skill and reputation during electives, and dressing well for interviews. Electives abroad, multiple recommendation letters, and being on the dean’s honor list were less important. Interview was the most favored domain (4.04 ± 0.78), followed by service and electives (4.01 ± 0.47); recommendations were the least favored (3.41 ± 0.77). While differences existed among variables, they weren’t statistically significant, except for the interview domain (*p =* 0.015), where experienced PDs relied more on interviews compared to first-time PDs.

**Conclusion:**

PDs showed diverse responses to all evaluated factors. Interviews and service/electives were most preferred. Service residencies, skills/reputation during electives, and being well dressed during interviews were favored for candidate selection. However, the results should be interpreted cautiously, given the potential limitations of statistical power associated with relatively small sample size.

## 1 Introduction

Dental caries is a significant public health concern and one of the most prevalent chronic diseases globally. In Saudi Arabia, the incidence of dental caries is high in both pediatric and adult populations, with studies estimating a prevalence of approximately 80% (1). Despite this high prevalence, the utilization of dental services remains suboptimal owing to various barriers, including prolonged wait times, dental anxiety, a perceived lack of need for care, and the financial burden of treatment, among others (2). Addressing these challenges and promoting preventive dental care have become priorities in Saudi Arabia. This commitment is encapsulated in the National Transformation Program (NTP), which aligns with Vision 2030 to enhance the public healthcare sector and diversify the economy (3). The dentist-to-patient ratio and the number of trained dental professionals graduating in Saudi Arabia are gradually improving, reflecting a growing interest in postgraduate education among dental students (4, 5). Medical and dental institutions are adapting to evolving healthcare demands and patient expectations while accommodating the diverse backgrounds of students by incorporating innovative teaching methodologies (6). The Saudi Commission for Health Specialties (SCFHS) is actively monitoring these developments and has accredited up to 21 master’s degree programs in the field of dentistry (7).

The Saudi Board in Restorative Dentistry (SBRD), now referred to as the Saudi Specialty Certificate in Restorative Dentistry [SSC-(Resto)], represents a favored pathway for many dental graduates, boasting the highest enrollment of dentistry residents as of 2023 (6). The program has integrated the CanMEDS 2015 framework to define core competencies for residents, with the objective of enhancing patient care (6, 8). The SBRD program encompasses a rigorous 3-year curriculum combining didactic instruction and clinical training, aiming to elevate dental standards in Saudi Arabia by equipping dentistry residents with the necessary skills to excel as restorative dentists. The program comprises 2 distinct phases: supervised junior residency and independent senior residency (9).

The oversight of the residency program in Saudi Arabia is administered through multiple levels of authority, with program directors (PDs) playing a pivotal role in the selection of candidates (10). The evaluation process allocates 55% of the total score to the Saudi Licensing Examination (SDLE) and 30% to the grade point average (GPA). The remaining 15% is distributed across various domains, including research, service/electives, interviews, and letters of recommendation (11). However, the specific importance assigned to these domains and the precise inquiries prioritized by PDs who are in the process of selecting candidates remain ambiguous. Gaining insight into these priorities is crucial for prospective candidates aiming to target key areas that may enhance their prospects of acceptance (12).

Previous investigations in Saudi Arabia on this topic have focused on the medical sector, exploring the views of PDs in specialties such as plastic surgery, pediatrics, orthopedics, and urology (10–13). While research has been conducted in some dental specialties, such as orthodontics and periodontics, these studies have primarily been descriptive and often lack comparative analyses (14, 15). Furthermore, they have tended to focus on a limited range of factors related to program selection, such as geographical region and professional experience (15). Given the significant variation in requirements and expectations across specialty program selection processes, the growing popularity of the SBRD program, and the lack of prior studies on this topic, this study aims to fill that gap by examining PDs’ perspectives on the residency candidate selection process for the SBRD program. It also aimed to compare these views based on the PD’s sex, current position, years of experience, across hospital settings, and geographic locations within the Kingdom of Saudi Arabia. This study offers a comprehensive analysis across these key PD characteristics, filling a critical gap and enhancing understanding of candidate selection in SBRD programs.

## 2 Materials and methods

### 2.1 Study design and ethical approval

This study employed a cross-sectional design study to collect data from PDs involved in the SBRD program. The Standing Committee for Bioethics Research at Prince Sattam Bin Abdulaziz University (approval number SCBR-425/2025; dated 9/2/2025) approved this study which was conducted in accordance with the Helsinki Declaration. Informed statement was obtained from each participant after receiving the Bioethics approval and only those who acknowledged participation were directed to the survey. A statement emphasizing the maintenance of anonymity with the assurance that no identifiable information was collected was provided.

### 2.2 Survey instrument and data collection

A slightly modified version of the previously validated survey instrument adopted from a study by Alheraisi et al. (14) to PDs via the SCFHS platform using Qualtrics (Qualtrics LLC, Utah, United States). All items were kept unchanged, except for one in the examination domain that investigated how specialty-specific grades in SDLE affect candidate selection. This item was omitted because it was deemed non-applicable, as the declared results are generalized and do not include individual specialty marks in the SDLE final results. The SBRD program comprises 43 officially recognized centers, listed on the SCFHS website.^1^ Participants in the survey were selected via convenience sampling, and selection bias was addressed by comparing responses with known SBRD center parameters provided on the website. The survey was conducted over a 3-month period, from February 15, 2025, to May 15, 2025, with 2 reminders. Inclusion criteria included restorative dentists who are or have worked previously as PDs at centers approved by SCFHS.

### 2.3 Questionnaire

The survey included 30 questions across seven domains. Participants who consented to participation proceeded to the demographic section consisting of the following five questions: 1. work status (current or former PD); 2. experience as PD (<1 year, 1–3 years, and more than 3 years); 3. sex (male/female), 4. hospital setting (university, Ministry of Health (MOH), military, private, general); and 5. geographic location (north, south, east, west, central Saudi Arabia).

The next section of the questionnaire examined the views of the PDs on residency program candidate characteristics. The first four questions assessed the opinions about the importance of GPA and undergraduate studies, focusing on the value of GPA, honors earned during graduate school, the reputation of the graduating dental school, and being on the dean’s honorary list. Two questions addressed issues related to examinations, specifically the SDLE scores and the value of passing international examinations such as the Integrated National Board Dental Examination (INBDE). The following 6 questions explored the research domain: evaluating the perceptions of research volume and quality, the reputation of the journals publishing their work, the alignment of their research with restorative dentistry, and any oral poster presentations in which they had participated.

The interview domain included the following three questions: the weight of interviews relative to the GPA and SDLE scores, the importance of professional appearance during interviews, and the assessment of the candidates’ knowledge of restorative dentistry. The services and electives domain comprised six questions which included the impact of choosing restorative dentistry electives during internships, the effect of working as a service resident, the influence of taking electives under renowned restorative dentists, electives taken abroad, and the influence of applicants’ reputations and skills during electives.

The recommendation letter domain included seven questions that evaluated the perceived importance of recommendation letters, value of multiple recommendations, influence of endorsements from restorative dentists, significance of personally knowing the recommender, reputation of the recommender, and whether the mode of recommendation affected selection outcomes. Additionally, two questions in the miscellaneous domain examined how community services and English language proficiency influenced the selection decisions. A 1–5 Likert scale was used to assess the data, with one indicating strongly disagree, two indicating disagree, three neither agree nor disagree, four agree, and five strongly agree.

### 2.4 Statistical analysis

SPSS 2024 software was used to analyze the data and calculate the frequencies and percentages of the demographic variables. The means and standard deviations for all questions and respective domains were computed to provide a comprehensive understanding of the perspectives of the PDs. Normality of the data was assessed using Shapiro-Wilk and Kolmogorov-Smirnov tests, which indicated that the data were normally distributed. In accordance with evidence supporting the use of parametric tests for Likert-type measures, Student’s *t*-test was used to compare mean scores by sex and work status among the PDs, whereas analysis of variance (ANOVA) was used to assess mean scores among the PDs across experience levels, hospital settings, and geographic locations (16).

## 3 Results

### 3.1 Characteristics of participants

**FIGURE 1:**
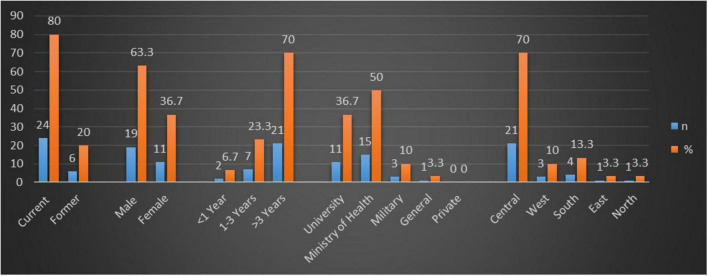
Graphical representation of program director characteristics.

A total of 62.5% of the distributed surveys were completed, resulting in 30 responses from an outreach to 48 PDs. Among the respondents, 80% were currently serving as PDs, whereas the remaining 20% were former PDs. The sex distribution showed that 63.3% of the respondents were male and 36.7% were female. Seventy percent of the respondents had more than 3 years of experience. A total of 23.3% had 1–3 years of experience, and 2 PDs were in their first year of service. Regarding hospital settings, 50% of the respondents were affiliated with MOH centers, 36.7% with university-affiliated centers, and 10% with military hospital centers. One response came from a general hospital and there were no responses from private centers. Regarding geographics, most respondents were from the central region (70%), followed by the southern region (13.3%) and the western region (10%). The northern and eastern regions were underrepresented, with each region contributing only one respondent. A graphical representation of these results is shown in Figure 1.

### 3.2 PDs’ response based on individual queries

The mean scores indicated that PDs assigned minimal importance to the mode of recommendation, whether by phone or written letter (mean, 3.07 ± 1.112), followed by the pursuit of electives abroad (mean, 3.20 ± 0.925) (Tables 1, 2). Similarly, the need for multiple recommendations and being on the dean’s honorary list earned a lower mean score of 3.27. Conversely, factors such as an applicant’s reputation and performance during electives among staff, previous work as service residents, and professional appearance (well dressed) during the interviews received the highest mean score of 4.40. Additionally, respondents valued candidates who had completed elective rotations in restorative dentistry during their internships, and those candidates having good SDLE scores achieved a mean score of 4.27.

**TABLE 1:** Means and standard deviations of GPA, exams, research, and interviews domains based on Likert scale responses.

Domains with individual queries	Strongly disagree	Disagree	Neutral	Agree	Strongly agree	Mean ± SD
**I. GPA**						**3.55 ± 0.63**
1. Does the grade point average (GPA) improve the chance of getting accepted?	1 (3.3)	2 (6.7)	6 (20)	14 (46.7)	7 (23.3)	3.80 ± .997
2. Does being on the Dean’s honorary list improve the chance of acceptance?	1 (3.3)	6 (20)	10 (33.3)	10 (33.3)	3 (10)	3.27 ± 1.015
3. Do candidates who gained awards or honors have a better chance of getting accepted in your program?	1 (3.3)	2 (6.7)	9 (30)	15 (50)	3 (10)	3.57 ± .898
4. Does the reputation of the medical school from which the applicant graduated influence their chance of acceptance into your program?	1 (3.3)	5 (16.7)	6 (20)	12 (40)	6 (20)	3.57 ± 1.104
**II. Exams**						**3.82 ± 0.74**
5. Is the candidate’s Saudi Dental License Exam (SDLE) score important?	1 (3.3)	0 (0)	2 (6.7)	14 (46.7)	13 (43.3)	4.27 ± .868
6. Does a candidate who passed international licensing examinations, such as the United States National Board Dental Examination (NBDE), have a better chance of being accepted in your program?	1 (3.3)	5 (16.7)	10 (33.3)	10 (33.3)	4 (13.3)	3.37 ± 1.033
**III. Research**						**3.73 ± 0.64**
7. Is having a general experience in research important? (By experience we mean evidence of knowledge in research through courses in medical research and publications).	0	0	4 (13.3)	23 (76.7)	3 (10)	3.97 ± .490
8. Is the quantity of publications important? (Regardless of whether they are in Restorative Dentistry or not).	1 (3.3)	4 (13.3)	9 (30)	12 (40)	4 (13.3)	3.47 ± 1.008
9. Is quality of publications important? (Regarding proper design, execution, and writing quality).	0	3 (10)	6 (20)	10 (33.3)	11 (36.7)	3.97 ± .999
10. Is publishing in prestigious journals important?	0	1 (3.3)	8 (26.7)	12 (40)	9 (30)	3.97 ± .850
11. Are publications in restorative dentistry the most important?	1 (3.3)	8 (26.7)	5 (16.7)	8 (26.7)	8 (26.7)	3.47 ± 1.252
12. Does presenting posters or oral presentations in events improve the chance of acceptance?	1 (3.3)	3 (10)	8 (26.7)	15 (50)	3 (10)	3.53 ± .937
**IV. Interview**						**4.04 ± 0.78**
13. Does the Interview has same weight as the SDLE score and the GPA?	1 (3.3)	7 (23.3)	1 (3.3)	9 (30)	12 (40)	3.80 ± 1.297
14. Do you prefer candidates who are well dressed during the interview?	0	1 (3.3)	2 (6.7)	11 (36.7)	16 (53.3)	4.40 ± .770
15. Do you look for the level of knowledge in Restorative Dentistry during the interview?	0	3 (10)	5 (16.7)	13 (43.3)	9 (30)	3.93 ± .944

Likert scale: Strongly disagree 1; Disagree 2; Neither agree nor disagree 3; Agree 4; Strongly agree 5. Bold values indicate the domain-level aggregate mean/standard deviation.

**TABLE 2:** Means and standard deviations of services and electives, recommendations, and miscellaneous domains based on Likert scale responses.

Domains with individual queries	Strongly disagree	Disagree	Neutral	Agree	Strongly agree	Mean ± SD
**I. Services and Electives**						**4.01 ± 0.47**
16. Is taking Restorative dentistry elective rotations during internship important?	1 (3.3)	1 (3.3)	1 (3.3)	13 (43.3)	14 (46.7)	4.27 ± .944
17. Does working as a service resident improve the chance of accepting an applicant?	1 (3.3)	0	1 (3.3)	12 (40)	16 (53.3)	4.40 ± .855
18. Candidates who worked/took electives in your department have a better chance of getting accepted?	1 (3.3)	1 (3.3)	7 (23.3)	9 (30)	12 (40)	4.00 ± 1.050
19. Does the applicant’s reputation and performance (during clinics or between staff) influence their chance of acceptance?	0	0	2 (6.7)	14 (46.7)	14 (46.7)	4.40 ± .621
20. Do candidates who have worked/taken electives abroad have a better chance of getting accepted?	2 (6.7)	2 (6.7)	16 (53.3)	8 (26.7)	2 (6.7)	3.20 ± .925
21. Do the candidates who worked/took electives with a distinguished restorative dentist have a better chance of getting accepted?	1 (3.3)	0	7 (23.3)	18 (60)	4 (13.3)	3.80 ± .805
**II. Recommendations**						**3.41 ± 0.77**
22. Is having a recommendation letter important?	1 (3.3)	6 (20)	7 (23.3)	12 (40)	4 (13.3)	3.40 ± 1.070
23. Is having multiple recommendations important?	2 (6.7)	4 (13.3)	12 (40)	8 (26.7)	4 (13.3)	3.27 ± 1.081
24. Are recommendations from a restorative dentist important?	2 (6.7)	1 (3.3)	7 (23.3)	15 (50)	5 (16.7)	3.67 ± 1.028
25. Is personally knowing the recommending person important?	3 (10)	3 (10)	10 (33.3)	9 (30)	5 (16.7)	3.33 ± 1.184
26. Is the reputation of the recommending person important?	2 (6.7)	1 (3.3)	5 (16.7)	17 (56.7)	5 (16.7)	3.73 ± 1.015
27. Is the mode of recommendation, such as phone calls or written letters, important?	4 (13.3)	4 (13.3)	9 (30)	12 (40)	1 (3.3)	3.07 ± 1.112
28. Is the quality of the language and content of the recommendation important?	2 (6.7)	4 (13.3)	9 (30)	9 (30)	6 (20)	3.43 ± 1.165
**III. Miscellaneous**						**3.67 ± 0.75**
29. Does participating in community service activities and volunteering improve the chance of acceptance?	2 (6.7)	3 (10)	8 (26.7)	16 (53.3)	1 (3.3)	3.37 ± .964
30. Is being fluent in English important?	2 (6.7)	0	2 (6.7)	19 (63.3)	7 (23.3)	3.97 ± .964

Likert scale: Strongly disagree 1; Disagree 2; Neither agree nor disagree 3; Agree 4; Strongly agree 5. Bold values indicate the domain-level aggregate mean/standard deviation.

### 3.3 PDs response based on domains

The domain analysis revealed that the candidate interview was rated as the most important by the PDs, with a mean of 4.04 ± 1.06. Likewise, the services and electives domains were also preferred, with a mean score of 4.01 ± 0.47. Conversely, PDs rated the recommendations domain the lowest, with a mean score of 3.41 ± 0.77. The GPA domain also received lower ratings, with a mean of 3.55 ± 0.63 (Tables 1, 2).

### 3.4 PDs response based on geographic location

The ANOVAs for the findings based on geographical region, hospital setting, and PD experience levels are shown in Tables 3–5. The results, as shown in Table 3, indicate variability among the groups; however, these differences were not statistically significant. PDs from the southern region exhibited higher mean scores in the domains of examinations (4 ± 0.40), research (4.13 ± 0.47), interviews (4.50 ± 0.43), and electives (4.38 ± 0.16) than their counterparts from other regions. Conversely, PDs from the central region showed higher mean scores for recommendations (3.40 ± 0.69) and the miscellaneous domain (3.74 ± 0.76). In the western region, PDs placed greater emphasis on GPA, with a mean score of 3.75 ± 0.43.

**TABLE 3:** Means and standard deviations of PD responses, along with an analysis of variance based on their geographical location.

Domains	Location	n	Mean	SD	*F*	*p*
GPA	Central	21	3.60	0.70	1.017	0.376 ns
	West	3	3.75	0.43		
	Southern	4	3.13	0.43		
Exams	Central	21	3.83	0.85	0.351	0.707 ns
	West	3	3.50	0.50		
	Southern	4	4.00	0.40		
Research	Central	21	3.71	0.64	1.472	0.249 ns
	West	3	3.28	0.91		
	Southern	4	4.13	0.47		
Interview	Central	21	3.98	0.88	0.671	0.520 ns
	West	3	4.00	0.57		
	Southern	4	4.50	0.43		
Electives	Central	21	3.88	0.49	2.298	0.121 ns
	West	3	4.17	0.16		
	Southern	4	4.38	0.16		
Recommendation	Central	21	3.40	0.69	0.089	0.915 ns
	West	3	3.19	0.64		
	Southern	4	3.39	1.42		
Miscellaneous	Central	21	3.74	0.76	0.343	0.713 ns
	West	3	3.33	0.57		
	Southern	4	3.63	1.10		

*n*, total number of participants; *F*, Analysis of variance (ANOVA) value; *p*, *p*-value with significance at < 0.05; ns, non-significant difference.

**TABLE 4:** Means and standard deviations of PD responses, along with an analysis of variance based on their hospital type.

Domains	Hospital type	n	Mean	SD	*F*	*p*
GPA	University	11	3.64	0.82	0.497	0.614 ns
	MOH	15	3.40	0.42		
	Military	3	3.50	0.00		
Exams	University	11	3.86	1.00	0.131	0.878 ns
	MOH	15	3.73	0.41		
	Military	3	3.67	1.04		
Research	University	11	3.80	0.78	0.079	0.924 ns
	MOH	15	3.71	0.61		
	Military	3	3.67	0.44		
Interview	University	11	3.73	1.00	1.394	0.266 ns
	MOH	15	4.24	0.54		
	Military	3	4.00	0.88		
Electives	University	11	3.85	0.56	1.207	0.315 ns
	MOH	15	4.07	0.40		
	Military	3	4.28	0.48		
Recommendation	University	11	3.48	0.56	0.471	0.630 ns
	MOH	15	3.26	0.95		
	Military	3	3.67	0.21		
Miscellaneous	University	11	4.00	0.59	2.245	0.126 ns
	MOH	15	3.40	0.82		
	Military	3	3.50	0.50		

n, total number of participants; F, Analysis of variance (ANOVA) value; *p*, *p*-value with significance at < 0.05. ns, non-significant difference.

**TABLE 5:** Means and standard deviations of PD responses, along with an analysis of variance based on their years of experience as PDs.

Domains	Years of experience	n	Mean	SD	F	*P*
GPA	0–1	2	3.50	0.35	0.608	0.552 ns
	1–3	7	3.79	0.96		
	3	21	3.48	0.53		
Exams	0–1	2	3.75	0.35	0.260	0.773 ns
	1–3	7	3.64	1.14		
	3	21	3.88	0.63		
Research	0–1	2	3.33	0.23	0.402	0.673 ns
	1–3	7	3.71	0.77		
	3	21	3.77	0.63		
Interview	0–1	2	3.17	0.70	4.969	0.015*
	1–3	7	3.52	0.99		
	3	21	4.30	0.57		
Electives	0–1	2	3.75	1.29	1.141	0.334 ns
	1–3	7	3.83	0.30		
	3	21	4.10	0.43		
Recommendation	0–1	2	3.71	0.20	0.236	0.792 ns
	1–3	7	3.29	0.63		
	3	21	3.43	0.85		
Miscellaneous	0–1	2	3.00	0.70	1.992	0.156 ns
	1–3	7	4.07	0.53		
	3	21	3.60	0.78		

n, total number of participants; F, Analysis of variance (ANOVA) value; *p*, *p*-value with significance at ≤ 0.05. * Significant difference with *p* ≤ 0.05

### 3.5 PDs response based on hospital setting

Additionally, PDs affiliated with university centers demonstrated higher means in the domains of GPA (3.64 ± 0.82), examinations (3.86 ± 1), research (3.80 ± 0.78), and miscellaneous (4 ± 0.59) than PDs from other centers (Table 4). PDs from MOH centers preferred interviews, with a mean score of 4.24 ± 0.54, while those from military centers showed higher mean scores in electives (4.28 ± 0.48) and recommendations (3.67 ± 0.21). Owing to underrepresentation, responses from the northern region and general hospitals were excluded from the analysis. In addition, no data was available from the eastern region or private hospitals.

### 3.6 PDs response based on experience

PDs with over 3 years of experience had higher means in the domains of examinations (3.88 ± 0.63), research (3.77 ± 0.63), interviews (4.30 ± 0.57), and electives (4.10 ± 0.43). In contrast, PDs with 1–3 years of experience showed higher means in GPA (3.79 ± 0.96) and miscellaneous (4.07 ± 0.53) domains. First-time PDs with < 1 year of experience relied more on the recommendation domain, with a mean score of 3.71 ± 0.20. A statistically significant difference was found in the interview domain; less experienced PDs (mean 3.17 ± 0.70) relied less on this resource than their more experienced counterparts (mean 4.30 ± 0.57) (Table 5).

### 3.7 PDs response based on their sex

Table 6 shows the results of the Student’s *t*-test, which compared the perspectives of PDs based on sex and work status. Although differences were noted across the seven assessed domains, these were not statistically significant. Males exhibited higher mean scores in the domains of GPA (3.58 ± 0.50), research (3.78 ± 0.65), interview (4.09 ± 0.71), and electives (4.11 ± 0.53) than females. Conversely, female PDs demonstrated superior mean scores in the domains of recommendations (mean 3.52 ± 0.51) and miscellaneous (mean 3.77 ± 0.78) relative to male PDs.

**TABLE 6:** Means and standard deviations of PD responses, along with Student’s *t*-test based on their work status and gender.

Domains	Position	n	Mean	Std. dev	*T*	*P*	Gender	n	Mean	SD	*t*	*p*
GPA	Current PD	24	3.57	0.68	0.388	0.701 ns	Male	19	3.58	0.50	0.322	0.750 ns
	Former PD	6	3.46	0.43			Female	11	3.50	0.84		
Exams	Current PD	24	3.85	0.78	0.542	0.592 ns	Male	19	3.82	0.65	0.008	0.992 ns
	Former PD	6	3.67	0.60			Female	11	3.82	0.92		
Research	Current PD	24	3.71	0.67	0.325	0.748 ns	Male	19	3.78	0.65	0.583	0.564 ns
	Former PD	6	3.81	0.58			Female	11	3.64	0.65		
Interview	Current PD	24	3.99	0.80	0.808	0.426 ns	Male	19	4.09	0.71	0.390	0.699 ns
	Former PD	6	4.28	0.71			Female	11	3.97	0.93		
Electives	Current PD	24	3.97	0.50	0.897	0.377 ns	Male	19	4.11	0.53	1.460	0.156 ns
	Former PD	6	4.17	0.33			Female	11	3.85	0.31		
Recommendation	Current PD	24	3.38	0.85	0.548	0.588 ns	Male	19	3.35	0.90	0.558	0.581 ns
	Former PD	6	3.57	0.31			Female	11	3.52	0.51		
Miscellaneous	Current PD	24	3.60	0.79	0.900	0.376 ns	Male	19	3.61	0.75	0.576	0.569 ns
	Former PD	6	3.92	0.58			Female	11	3.77	0.78		

n, total number of participants; t, Student’s *t*-test; *p*, *p*-value with significance at ≤ 0.05. *Significant difference with *p* ≤ 0.05.

### 3.8 PDs response based on their work status

Additionally, PDs currently serving in their positions showed higher mean scores in the domains of GPA (3.57 ± 0.68) and examinations (3.85 ± 0.78) than former PDs. In contrast, former PDs had higher mean scores in the domains of research (3.81 ± 0.58), interviews (4.28 ± 0.71), electives (4.17 ± 0.33), recommendations (3.57 ± 0.31), and miscellaneous (3.92 ± 0.58).

## 4 Discussion

This study was conducted to evaluate the perspectives of PDs on candidate selection for an SBRD program. To our knowledge, this is the first study in KSA to focus on restorative PDs. Previous research has primarily focused on periodontic and orthodontic PDs in dentistry, as well as on PDs in other medical programs. The findings of this study revealed that interviews were the most favored domain among the PDs, followed by services and electives. Specifically, individual queries revealed higher preferences for candidates’ performance and reputation during electives, having worked as service residents, and being well dressed during interviews. Conversely, factors such as pursuing electives abroad, the number and mode of recommendation letters, and being on the dean’s honorary list did not positively influence the perceptions of the PDs. These trends remained consistent across the various PD-related factors examined in this study, except in the interview domain, where responses varied according to PD experience level.

The lower average values of the GPA and examinations domains compared with other domains in this study may have resulted from the fact that the GPA and SDLE had predefined weights. The selection process for the SCFHS Board programs assigns 55% importance to the SDLE examinations, 30% to the GPA, and the remaining 15% to other factors (12). Assuming all candidates reached the next stage with good GPA and SDLE scores, the PDs likely concentrated on other aspects of the selection process. A similar finding was reported for plastic surgery PDs in Saudi Arabia (10). However, specific elements within these domains, such as the significance of the graduating school, being on the dean’s honorary list, awards earned during dental school, and the influence of passing international examinations, required further exploration and were therefore included in the survey. GPA provides an overall assessment of graduate academic performance; however, some studies have focused specifically on clinical grades during dental school (17). The SBRD emphasizes clinical competencies; therefore, it is plausible that greater weight is placed on clinical performance than overall GPA. The lower average scores in these domains may also be due to lower scores on individual questions, such as being on the dean’s honorary list and passing international examinations, which affected the domains’ total scores despite the high importance placed on the SDLE examinations. A previous study on PDs for the Saudi Board Pediatric program ranked the Saudi Medical Licensing Exam (SMLE) as the highest priority (11). The SDLE and its counterpart, the SMLE, serve as licensing examinations for practicing dentistry and medicine in Saudi Arabia, underscoring the significance that PDs attach to these assessments. A reasonable explanation for this strong appeal is that licensing is essential for clinical work throughout the programs, and those who achieve strong scores demonstrate a sufficient level of knowledge and ability to excel in competitive examinations.

Research experience is vital for strengthening a candidate’s application to dental residency programs (18). A solid understanding of oral biology relies on foundational knowledge, research skills, and appreciation for research, which often play a key role in postgraduate admissions (19, 20). Despite growing interest in research-based postgraduate education, programs with a dedicated research unit tend to emphasize research more than those with a primary clinical focus (21). In this study, consistent with findings from various dental programs, the aggregate score for the research domain was lower than that for other domains (14, 17). Nevertheless, individual factors, such as the significance of basic research knowledge, were prioritized. Furthermore, PDs placed emphasis on the quality of candidates’ research and the prestige of journals over mere publication output, as reflected in their responses. A possible reason could be their understanding that publishing in esteemed journals demands high-quality research and considerable investment in time and effort (22).

In this study, the interview domain emerged as the most popular choice for restorative PDs, followed by services and electives. Interviews remain an integral part of the selection process; however, the academic results may not necessarily match the interview ratings (23). The candidate’s choice of professional attire during the interview was among the most significant factors influencing PDs in the present study. This finding is consistent with results from a study of Saudi pediatric PDs, in which the most important element of interviews was the ability of the candidates to present themselves well through appropriate dress (11). Conversely, Saudi urology PDs placed less emphasis on this aspect (13). The impact of being well dressed is twofold: it enhances self-confidence and creates a positive impression on the interviewer. Conversely, those dressed unprofessionally are often perceived negatively, particularly within a noble profession such as dentistry (24). In studies focusing on periodontic and orthodontic PDs in the United States, the interview process had consistently emerged as a pivotal criterion for candidate selection (17, 25). Interviews provide a valuable opportunity to assess students’ knowledge and expertise in restorative dentistry, as evidenced by the higher means observed in this study. In plastic surgery, orthopedics, and urology programs in Saudi Arabia, PDs placed significant emphasis on their personal interactions with candidates during the interview process (10, 12, 13). These interactions helped deepen their understanding of the candidates’ knowledge and capabilities.

The importance placed on candidates who completed electives abroad was minimal in this study, which aligns with previous research (13, 14). This may be attributed to a lack of understanding of and connection with the international centers where these electives have occurred, which subsequently constrained the ability to obtain valuable feedback on the candidates’ performances. Conversely, candidates who worked as service residents in the restorative field and demonstrated strong clinical skills and positive rapport with staff were the most appealing to PDs among the other domains. This preference likely indicates a demonstrated commitment, signifying a clear dedication to pursuing a postgraduate degree in restorative dentistry (11, 13, 14). Given the significance of this result, it should remain the primary focus of aspiring candidates.

Contrary to previous studies that underscored the importance of recommendation letters in the evaluation process, we identified the domain of recommendations as the least prioritized by the restorative PDs. In a study of oral surgery PDs in the United States, Laskin et al. found that 90% of the respondents agreed on the importance of recommendation letters in candidate selection, particularly those received from university faculty (26). Notably, in our study, the mode of recommendations and the requirement for multiple letters received the lowest ratings, adversely affecting the overall average for this domain. The oral surgery PDs in the United States study preferred letters over recommendation forms, reporting that letters would provide more information, suggesting a possible review of the content and language of the letter (26). A study of pediatric PDs in Saudi Arabia reported similar findings to ours, indicating minimal emphasis on recommendation letters in their assessment criteria (11). However, recommendations hold particular weight when sourced from reputable individuals within the same profession. Other significant factors, although often considered supplementary, are candidates’ English language proficiency and, to a lesser degree, their involvement in community services. Previous research has also highlighted the importance of English language skills in candidate evaluation (14).

New PDs with < 1 year of experience tended to rely more on recommendation letters and less on interviews than their more experienced counterparts, resulting in a statistically significant difference. Notably, the difference in means was particularly pronounced in the responses concerning the value of the interview along with the GPA, where less experienced PDs tended to respond negatively. This may be attributed to their reliance on external recommendations rather than independent evaluations of candidates during the interview process. A previous study on Saudi urology PDs identified significant differences in experience levels regarding queries about recommendation letters from non-urologists (13). Although differences were noted in other queries involving PD variables in the study, they were not statistically significant.

Our study had several limitations that should be considered when interpreting the results. First, the small sample size and the use of convenience sampling may introduce selection bias, which could limit the generalizability of our findings. Second, variations in responses across different variables within each domain may have influenced the results. Despite these limitations, we believe that the findings of this study hold significant value. Furthermore, this is the first study to examine PDs’ perspectives on candidates for restorative dentistry programs. Our findings could be especially useful for prospective candidates, given the strong demand for restorative dental courses in Saudi Arabia. The outcomes of the study should be regarded as specific to the SBRD. Nevertheless, these selection dynamics may be relevant to other board programs in dentistry and medicine in Saudi Arabia that operate under comparable frameworks established by SCFHS. However, they may not generalize fully to other or international training systems, as evaluation criteria can vary. To distinguish among candidates, programs should adopt standardized, competency-based interview frameworks that include structured scoring rubrics to reduce the inherent subjectivity in these assessments. Additionally, formalizing assessment criteria for service residency and elective performance, such as standardized evaluation forms or minimum competency checklists, may also help reduce variability among programs. Ultimately, such results would assist programs in refining their selection processes to better identify and attract the most suitable candidates.

## 5 Conclusion

In conclusion, while all components of the evaluation process carried varying degrees of significance, PDs placed particular emphasis on candidates’ skills and performance during electives and on those who worked as service residents. Furthermore, the professional appearance of the candidates during the interview positively influenced the perceptions of the PDs. Given the minimal variability observed among the PD-related variables examined in this study, we can infer a consistent selection pattern across the SBRD program.

## Data Availability

The original contributions presented in this study are included in this article/supplementary material, further inquiries can be directed to the corresponding author.

## Footnotes

1
https://scfhs.org.sa/en/training?tid=30
